# Bis(*N*-benzoyl-*N*-phenyl­hydroxy­l­aminato-κ^2^ *O*,*O*′)dimethyl­tin(IV)

**DOI:** 10.1107/S160053681102561X

**Published:** 2011-07-06

**Authors:** Jin Jiang, Lei Dong, Handong Yin

**Affiliations:** a College of Chemistry and Chemical Engineering, Liaocheng University, Shandong 252059, People’s Republic of China

## Abstract

The Sn atom in the title compound, [Sn(CH_3_)_2_(C_13_H_10_NO_2_)_2_], has a highly distorted octa­hedral coordination with the equatorial plane made up of four O atoms from two *N*-benzoyl-*N*-phenyl­hydroxy­laminate ligands and the axial positions occupied by two methyl groups. The crystal structure is stabilized by van der Waals inter­actions.

## Related literature

For related structures, see: Harrison *et al.* (1976 ▶)
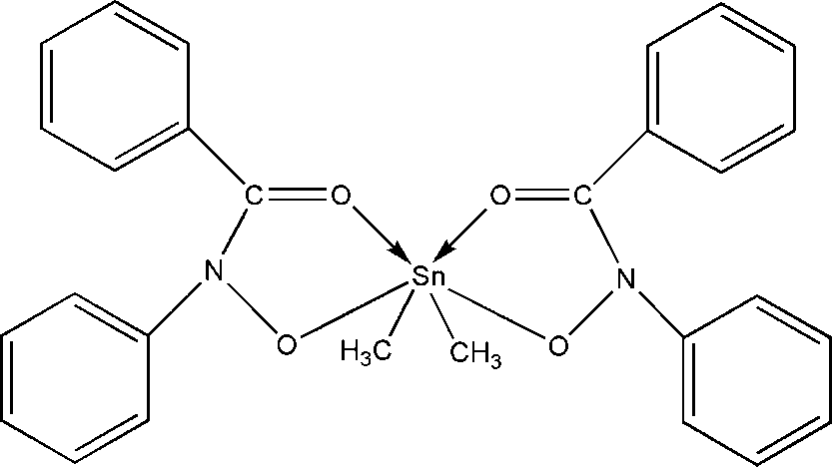


## Experimental

### 

#### Crystal data

[Sn(CH_3_)_2_(C_13_H_10_NO_2_)_2_]
*M* *_r_* = 573.20
Monoclinic, 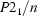

*a* = 13.5475 (14) Å
*b* = 10.3621 (11) Å
*c* = 19.0161 (19) Å
β = 102.128 (1)°
*V* = 2609.9 (5) Å^3^
*Z* = 4
Mo *K*α radiation
μ = 1.01 mm^−1^
*T* = 298 K
0.45 × 0.41 × 0.28 mm

#### Data collection

Bruker SMART CCD area-detector diffractometer
Absorption correction: multi-scan (*SADABS*; Sheldrick, 1996 ▶) *T*_min_ = 0.658, *T*_max_ = 0.764
13127 measured reflections
4617 independent reflections
2999 reflections with *I* > 2σ(*I*)
*R*_int_ = 0.054

#### Refinement

*R*[*F* ^2^ > 2σ(*F* ^2^)] = 0.042
*wR*(*F* ^2^) = 0.118
*S* = 1.12
4617 reflections
316 parameters
H-atom parameters constrained
Δρ_max_ = 1.34 e Å^−3^
Δρ_min_ = −0.65 e Å^−3^

### 

Data collection: *SMART* (Bruker, 2007 ▶); cell refinement: *SAINT* (Bruker, 2007 ▶); data reduction: *SAINT*; program(s) used to solve structure: *SHELXS97* (Sheldrick, 2008 ▶); program(s) used to refine structure: *SHELXL97* (Sheldrick, 2008 ▶); molecular graphics: *SHELXTL* (Sheldrick, 2008 ▶); software used to prepare material for publication: *SHELXTL*.

## Supplementary Material

Crystal structure: contains datablock(s) I, global. DOI: 10.1107/S160053681102561X/bx2356sup1.cif

Structure factors: contains datablock(s) I. DOI: 10.1107/S160053681102561X/bx2356Isup2.hkl

Additional supplementary materials:  crystallographic information; 3D view; checkCIF report

## Figures and Tables

**Fig. 1.**
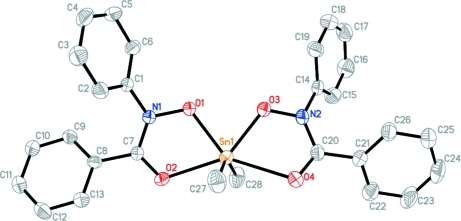
The molecular structure of the compound, showing 50% probability displacement ellipsoids. H atoms have been omitted for clarity.

## supplementary crystallographic information

### Comment

Among many multidentate organic ligands, hydroxamic acids are of particular importance, because of their remarkable structural diversity and biological applications. The molecular structure of the title compound is depicted in Fig.1. The Sn atom has a highly distorted octahedral coordination, with the equatorial plane made up of four O atoms of *N*-phenyl-*N*-benzoylhydroxylamino ligand and the axial positions occupied by a two methyl groups . The crystal structure is built up by van der Waals interactions.

### Experimental

The reaction was carried out under nitrogen atmosphere. *N*-phenylbenzohydroxamic acid (0.4 mmol) and KOH (0.4 mmol) were added to a stirred solution of methanol (30 ml) in a Schlenk flask for 0.5 h, then dimethyltin dichloride (0.2 mmol) was added to the reactor. The reaction mixture was stirred for 8 h at room temperature and then filtrated. The filtrate was evaporated *in vacuo* to dryness. The obtained solid was recrystallized from ethylether-petroleum ether (*v*/*v*, 1:1) (Yield 78%). Anal. Calcd (%) for C_28_H_26_N_2_O_4_Sn (Mr = 573.20): C, 58.67; H, 4.57; N, 4.89; O, 11.16. Found (%): C, 58.60; H, 4.51; N, 4.97; O, 11.08.

### Refinement

The C–H H atoms were positioned with idealized geometry and were refined isotropically with *U*_iso_(H) = 1.2 *U*_eq_(C) or 1.5 *U*_eq_(C).

### Crystal data

**Table:**

[Sn(CH_3_)_2_(C_13_H_10_NO_2_)_2_]	*F*(000) = 1160
*M**_r_* = 573.20	*D*_x_ = 1.459 Mg m^−^^3^
Monoclinic, *P*2_1_/*n*	Mo *K*α radiation, λ = 0.71073 Å
Hall symbol: -P 2yn	Cell parameters from 3950 reflections
*a* = 13.5475 (14) Å	θ = 2.2–23.5°
*b* = 10.3621 (11) Å	µ = 1.01 mm^−^^1^
*c* = 19.0161 (19) Å	*T* = 298 K
β = 102.128 (1)°	Block, colourless
*V* = 2609.9 (5) Å^3^	0.45 × 0.41 × 0.28 mm
*Z* = 4	

### Data collection

**Table:**

Bruker SMART CCD area-detector diffractometer	4617 independent reflections
Radiation source: fine-focus sealed tube	2999 reflections with *I* > 2σ(*I*)
graphite	*R*_int_ = 0.054
φ and ω scans	θ_max_ = 25.0°, θ_min_ = 1.7°
Absorption correction: multi-scan (*SADABS*; Sheldrick, 1996)	*h* = −9→16
*T*_min_ = 0.658, *T*_max_ = 0.764	*k* = −12→12
13127 measured reflections	*l* = −22→19

### Refinement

**Table:**

Refinement on *F*^2^	Primary atom site location: structure-invariant direct methods
Least-squares matrix: full	Secondary atom site location: difference Fourier map
*R*[*F*^2^ > 2σ(*F*^2^)] = 0.042	Hydrogen site location: inferred from neighbouring sites
*wR*(*F*^2^) = 0.118	H-atom parameters constrained
*S* = 1.12	*w* = 1/[σ^2^(*F*_o_^2^) + (0.0414*P*)^2^ + 2.3713*P*] where *P* = (*F*_o_^2^ + 2*F*_c_^2^)/3
4617 reflections	(Δ/σ)_max_ = 0.001
316 parameters	Δρ_max_ = 1.34 e Å^−^^3^
0 restraints	Δρ_min_ = −0.65 e Å^−^^3^

### Fractional atomic coordinates and isotropic or equivalent isotropic displacement parameters (Å^2^)

**Table:**

	*x*	*y*	*z*	*U*_iso_*/*U*_eq_	
Sn1	0.53023 (3)	0.21609 (4)	0.87352 (2)	0.04530 (15)	
N1	0.3263 (3)	0.1438 (4)	0.7933 (2)	0.0438 (11)	
N2	0.7002 (3)	0.2851 (5)	0.8049 (2)	0.0484 (11)	
O1	0.4223 (2)	0.1710 (4)	0.78095 (18)	0.0548 (11)	
O2	0.3965 (3)	0.1015 (4)	0.90754 (18)	0.0540 (10)	
O3	0.5963 (3)	0.2700 (4)	0.7884 (2)	0.0585 (11)	
O4	0.6905 (3)	0.3335 (4)	0.9179 (2)	0.0574 (10)	
C1	0.2476 (4)	0.1830 (5)	0.7352 (3)	0.0427 (14)	
C2	0.1697 (4)	0.2588 (6)	0.7472 (3)	0.0542 (16)	
H2	0.1660	0.2826	0.7937	0.065*	
C3	0.0970 (5)	0.2991 (6)	0.6893 (4)	0.0653 (18)	
H3	0.0437	0.3498	0.6972	0.078*	
C4	0.1015 (5)	0.2663 (7)	0.6210 (4)	0.0684 (19)	
H4	0.0514	0.2935	0.5827	0.082*	
C5	0.1807 (5)	0.1923 (6)	0.6088 (3)	0.0659 (19)	
H5	0.1854	0.1714	0.5621	0.079*	
C6	0.2531 (4)	0.1493 (6)	0.6665 (3)	0.0523 (15)	
H6	0.3057	0.0973	0.6586	0.063*	
C7	0.3195 (4)	0.1019 (5)	0.8581 (3)	0.0416 (13)	
C8	0.2221 (4)	0.0502 (5)	0.8709 (3)	0.0418 (13)	
C9	0.1598 (4)	−0.0267 (5)	0.8222 (3)	0.0477 (14)	
H9	0.1740	−0.0413	0.7771	0.057*	
C10	0.0757 (4)	−0.0825 (6)	0.8401 (3)	0.0545 (15)	
H10	0.0333	−0.1350	0.8073	0.065*	
C11	0.0555 (5)	−0.0599 (7)	0.9062 (4)	0.0704 (19)	
H11	−0.0014	−0.0969	0.9181	0.084*	
C12	0.1170 (5)	0.0159 (8)	0.9551 (4)	0.087 (2)	
H12	0.1019	0.0313	0.9999	0.104*	
C13	0.2012 (4)	0.0693 (6)	0.9380 (3)	0.0624 (17)	
H13	0.2447	0.1187	0.9719	0.075*	
C14	0.7473 (4)	0.2419 (5)	0.7494 (3)	0.0451 (14)	
C15	0.8264 (4)	0.1561 (6)	0.7627 (3)	0.0592 (16)	
H15	0.8509	0.1261	0.8092	0.071*	
C16	0.8692 (5)	0.1146 (7)	0.7071 (4)	0.073 (2)	
H16	0.9232	0.0573	0.7165	0.088*	
C17	0.8334 (5)	0.1565 (8)	0.6389 (4)	0.078 (2)	
H17	0.8633	0.1290	0.6017	0.093*	
C18	0.7536 (5)	0.2390 (7)	0.6253 (4)	0.074 (2)	
H18	0.7278	0.2654	0.5783	0.088*	
C19	0.7100 (4)	0.2843 (6)	0.6800 (3)	0.0586 (16)	
H19	0.6564	0.3423	0.6703	0.070*	
C20	0.7432 (4)	0.3252 (5)	0.8716 (3)	0.0479 (14)	
C21	0.8507 (4)	0.3625 (6)	0.8857 (3)	0.0497 (14)	
C22	0.9129 (5)	0.3195 (7)	0.9482 (4)	0.074 (2)	
H22	0.8871	0.2679	0.9802	0.089*	
C23	1.0133 (5)	0.3535 (8)	0.9630 (4)	0.091 (3)	
H23	1.0555	0.3248	1.0051	0.110*	
C24	1.0515 (5)	0.4296 (9)	0.9159 (5)	0.098 (3)	
H24	1.1196	0.4512	0.9261	0.117*	
C25	0.9905 (5)	0.4736 (7)	0.8546 (4)	0.077 (2)	
H25	1.0167	0.5256	0.8230	0.092*	
C26	0.8897 (4)	0.4407 (6)	0.8395 (3)	0.0605 (17)	
H26	0.8476	0.4714	0.7978	0.073*	
C27	0.4692 (5)	0.3809 (6)	0.9127 (4)	0.074 (2)	
H27A	0.4747	0.4532	0.8822	0.111*	
H27B	0.5057	0.3990	0.9607	0.111*	
H27C	0.3995	0.3659	0.9132	0.111*	
C28	0.6163 (4)	0.0528 (6)	0.9129 (4)	0.0716 (19)	
H28A	0.5905	−0.0212	0.8845	0.107*	
H28B	0.6123	0.0381	0.9620	0.107*	
H28C	0.6854	0.0667	0.9100	0.107*	

### Atomic displacement parameters (Å^2^)

**Table:**

	*U*^11^	*U*^22^	*U*^33^	*U*^12^	*U*^13^	*U*^23^
Sn1	0.0388 (2)	0.0544 (3)	0.0415 (2)	−0.00119 (19)	0.00566 (15)	0.0018 (2)
N1	0.035 (2)	0.060 (3)	0.036 (3)	−0.002 (2)	0.0069 (19)	0.004 (2)
N2	0.032 (2)	0.064 (3)	0.049 (3)	−0.002 (2)	0.009 (2)	0.004 (3)
O1	0.033 (2)	0.094 (3)	0.037 (2)	−0.009 (2)	0.0075 (16)	0.003 (2)
O2	0.046 (2)	0.076 (3)	0.036 (2)	−0.0099 (19)	−0.0016 (17)	0.0090 (19)
O3	0.033 (2)	0.097 (3)	0.044 (2)	−0.006 (2)	0.0044 (16)	0.011 (2)
O4	0.052 (2)	0.072 (3)	0.050 (3)	−0.009 (2)	0.0146 (19)	0.002 (2)
C1	0.033 (3)	0.055 (4)	0.039 (3)	−0.005 (2)	0.005 (2)	0.005 (3)
C2	0.050 (4)	0.062 (4)	0.052 (4)	0.005 (3)	0.014 (3)	0.004 (3)
C3	0.057 (4)	0.063 (5)	0.075 (5)	0.011 (3)	0.011 (3)	0.016 (4)
C4	0.051 (4)	0.079 (5)	0.066 (5)	−0.005 (4)	−0.009 (3)	0.020 (4)
C5	0.064 (4)	0.089 (5)	0.039 (4)	−0.014 (4)	−0.002 (3)	0.005 (3)
C6	0.044 (3)	0.068 (4)	0.043 (4)	0.005 (3)	0.006 (3)	−0.005 (3)
C7	0.035 (3)	0.050 (3)	0.040 (3)	−0.004 (2)	0.007 (2)	0.002 (3)
C8	0.043 (3)	0.050 (3)	0.032 (3)	0.004 (3)	0.009 (2)	0.002 (3)
C9	0.049 (3)	0.053 (4)	0.043 (3)	−0.005 (3)	0.014 (3)	−0.007 (3)
C10	0.048 (4)	0.062 (4)	0.052 (4)	−0.008 (3)	0.008 (3)	−0.003 (3)
C11	0.051 (4)	0.100 (6)	0.062 (4)	−0.018 (4)	0.019 (3)	−0.001 (4)
C12	0.075 (5)	0.135 (7)	0.061 (5)	−0.030 (5)	0.037 (4)	−0.026 (5)
C13	0.055 (4)	0.087 (5)	0.046 (4)	−0.012 (3)	0.012 (3)	−0.013 (3)
C14	0.031 (3)	0.053 (4)	0.050 (3)	−0.006 (2)	0.006 (2)	0.000 (3)
C15	0.058 (4)	0.057 (4)	0.059 (4)	0.005 (3)	0.005 (3)	0.003 (3)
C16	0.058 (4)	0.076 (5)	0.089 (6)	0.012 (4)	0.021 (4)	−0.009 (4)
C17	0.069 (5)	0.095 (6)	0.078 (6)	−0.015 (4)	0.034 (4)	−0.018 (5)
C18	0.075 (5)	0.096 (6)	0.051 (4)	−0.009 (4)	0.017 (4)	0.002 (4)
C19	0.048 (3)	0.074 (4)	0.055 (4)	0.008 (3)	0.013 (3)	0.012 (3)
C20	0.039 (3)	0.050 (4)	0.054 (4)	−0.002 (3)	0.010 (3)	0.007 (3)
C21	0.044 (3)	0.050 (4)	0.053 (4)	−0.002 (3)	0.006 (3)	−0.003 (3)
C22	0.056 (4)	0.087 (5)	0.071 (5)	−0.008 (4)	−0.002 (3)	0.011 (4)
C23	0.050 (4)	0.113 (6)	0.095 (6)	−0.009 (4)	−0.021 (4)	0.015 (5)
C24	0.042 (4)	0.126 (7)	0.118 (7)	−0.024 (4)	0.000 (4)	−0.003 (6)
C25	0.060 (4)	0.089 (5)	0.082 (5)	−0.021 (4)	0.013 (4)	0.003 (4)
C26	0.051 (4)	0.063 (4)	0.067 (4)	−0.008 (3)	0.011 (3)	−0.001 (3)
C27	0.067 (4)	0.065 (5)	0.097 (5)	−0.002 (3)	0.031 (4)	−0.009 (4)
C28	0.050 (4)	0.061 (4)	0.098 (5)	0.004 (3)	0.004 (3)	0.015 (4)

### Geometric parameters (Å, °)

**Table:**

Sn1—O3	2.083 (4)	C11—H11	0.9300
Sn1—O1	2.091 (3)	C12—C13	1.367 (8)
Sn1—C28	2.101 (6)	C12—H12	0.9300
Sn1—C27	2.101 (6)	C13—H13	0.9300
Sn1—O2	2.367 (4)	C14—C15	1.374 (8)
Sn1—O4	2.477 (4)	C14—C19	1.383 (8)
N1—C7	1.328 (6)	C15—C16	1.377 (9)
N1—O1	1.398 (5)	C15—H15	0.9300
N1—C1	1.425 (6)	C16—C17	1.358 (9)
N2—C20	1.345 (7)	C16—H16	0.9300
N2—O3	1.385 (5)	C17—C18	1.360 (9)
N2—C14	1.415 (7)	C17—H17	0.9300
O2—C7	1.249 (5)	C18—C19	1.382 (9)
O4—C20	1.247 (6)	C18—H18	0.9300
C1—C6	1.370 (7)	C19—H19	0.9300
C1—C2	1.373 (7)	C20—C21	1.476 (7)
C2—C3	1.379 (8)	C21—C22	1.379 (8)
C2—H2	0.9300	C21—C26	1.379 (8)
C3—C4	1.355 (9)	C22—C23	1.376 (9)
C3—H3	0.9300	C22—H22	0.9300
C4—C5	1.377 (9)	C23—C24	1.374 (10)
C4—H4	0.9300	C23—H23	0.9300
C5—C6	1.382 (7)	C24—C25	1.358 (10)
C5—H5	0.9300	C24—H24	0.9300
C6—H6	0.9300	C25—C26	1.379 (8)
C7—C8	1.491 (7)	C25—H25	0.9300
C8—C9	1.369 (7)	C26—H26	0.9300
C8—C13	1.378 (7)	C27—H27A	0.9600
C9—C10	1.382 (7)	C27—H27B	0.9600
C9—H9	0.9300	C27—H27C	0.9600
C10—C11	1.361 (8)	C28—H28A	0.9600
C10—H10	0.9300	C28—H28B	0.9600
C11—C12	1.362 (8)	C28—H28C	0.9600
			
O3—Sn1—O1	75.22 (13)	C11—C12—C13	119.5 (6)
O3—Sn1—C28	101.3 (2)	C11—C12—H12	120.2
O1—Sn1—C28	110.9 (2)	C13—C12—H12	120.2
O3—Sn1—C27	108.8 (2)	C12—C13—C8	120.4 (6)
O1—Sn1—C27	102.9 (2)	C12—C13—H13	119.8
C28—Sn1—C27	139.3 (3)	C8—C13—H13	119.8
O3—Sn1—O2	145.61 (13)	C15—C14—C19	119.7 (6)
O1—Sn1—O2	71.21 (13)	C15—C14—N2	121.6 (5)
C28—Sn1—O2	84.1 (2)	C19—C14—N2	118.7 (5)
C27—Sn1—O2	86.0 (2)	C14—C15—C16	120.0 (6)
O3—Sn1—O4	69.15 (13)	C14—C15—H15	120.0
O1—Sn1—O4	143.55 (13)	C16—C15—H15	120.0
C28—Sn1—O4	83.89 (19)	C17—C16—C15	120.7 (6)
C27—Sn1—O4	82.0 (2)	C17—C16—H16	119.7
O2—Sn1—O4	145.05 (12)	C15—C16—H16	119.7
C7—N1—O1	118.1 (4)	C16—C17—C18	119.5 (7)
C7—N1—C1	128.8 (4)	C16—C17—H17	120.3
O1—N1—C1	112.5 (4)	C18—C17—H17	120.3
C20—N2—O3	117.8 (4)	C17—C18—C19	121.3 (7)
C20—N2—C14	128.8 (4)	C17—C18—H18	119.3
O3—N2—C14	113.0 (4)	C19—C18—H18	119.3
N1—O1—Sn1	114.4 (3)	C18—C19—C14	118.8 (6)
C7—O2—Sn1	111.1 (3)	C18—C19—H19	120.6
N2—O3—Sn1	115.9 (3)	C14—C19—H19	120.6
C20—O4—Sn1	108.4 (3)	O4—C20—N2	119.2 (5)
C6—C1—C2	120.2 (5)	O4—C20—C21	122.9 (5)
C6—C1—N1	119.1 (5)	N2—C20—C21	117.8 (5)
C2—C1—N1	120.7 (5)	C22—C21—C26	119.5 (6)
C1—C2—C3	119.0 (6)	C22—C21—C20	118.2 (6)
C1—C2—H2	120.5	C26—C21—C20	122.3 (5)
C3—C2—H2	120.5	C23—C22—C21	119.5 (7)
C4—C3—C2	121.4 (6)	C23—C22—H22	120.3
C4—C3—H3	119.3	C21—C22—H22	120.3
C2—C3—H3	119.3	C24—C23—C22	120.4 (7)
C3—C4—C5	119.6 (6)	C24—C23—H23	119.8
C3—C4—H4	120.2	C22—C23—H23	119.8
C5—C4—H4	120.2	C25—C24—C23	120.5 (7)
C4—C5—C6	119.6 (6)	C25—C24—H24	119.7
C4—C5—H5	120.2	C23—C24—H24	119.7
C6—C5—H5	120.2	C24—C25—C26	119.5 (7)
C1—C6—C5	120.1 (6)	C24—C25—H25	120.2
C1—C6—H6	119.9	C26—C25—H25	120.2
C5—C6—H6	119.9	C25—C26—C21	120.6 (6)
O2—C7—N1	119.2 (5)	C25—C26—H26	119.7
O2—C7—C8	120.3 (5)	C21—C26—H26	119.7
N1—C7—C8	120.4 (4)	Sn1—C27—H27A	109.5
C9—C8—C13	119.5 (5)	Sn1—C27—H27B	109.5
C9—C8—C7	122.7 (5)	H27A—C27—H27B	109.5
C13—C8—C7	117.4 (5)	Sn1—C27—H27C	109.5
C8—C9—C10	120.0 (5)	H27A—C27—H27C	109.5
C8—C9—H9	120.0	H27B—C27—H27C	109.5
C10—C9—H9	120.0	Sn1—C28—H28A	109.5
C11—C10—C9	119.4 (5)	Sn1—C28—H28B	109.5
C11—C10—H10	120.3	H28A—C28—H28B	109.5
C9—C10—H10	120.3	Sn1—C28—H28C	109.5
C10—C11—C12	121.1 (6)	H28A—C28—H28C	109.5
C10—C11—H11	119.4	H28B—C28—H28C	109.5
C12—C11—H11	119.4		
			
C7—N1—O1—Sn1	−24.6 (6)	O2—C7—C8—C9	−137.2 (6)
C1—N1—O1—Sn1	147.3 (3)	N1—C7—C8—C9	40.0 (8)
O3—Sn1—O1—N1	−166.9 (4)	O2—C7—C8—C13	35.0 (8)
C28—Sn1—O1—N1	96.5 (4)	N1—C7—C8—C13	−147.7 (6)
C27—Sn1—O1—N1	−60.5 (4)	C13—C8—C9—C10	1.2 (8)
O2—Sn1—O1—N1	20.7 (3)	C7—C8—C9—C10	173.3 (5)
O4—Sn1—O1—N1	−154.5 (3)	C8—C9—C10—C11	0.2 (9)
O3—Sn1—O2—C7	−30.5 (5)	C9—C10—C11—C12	−0.5 (10)
O1—Sn1—O2—C7	−17.5 (4)	C10—C11—C12—C13	−0.7 (12)
C28—Sn1—O2—C7	−131.9 (4)	C11—C12—C13—C8	2.2 (11)
C27—Sn1—O2—C7	87.6 (4)	C9—C8—C13—C12	−2.4 (10)
O4—Sn1—O2—C7	157.6 (3)	C7—C8—C13—C12	−174.9 (6)
C20—N2—O3—Sn1	−31.1 (6)	C20—N2—C14—C15	45.2 (9)
C14—N2—O3—Sn1	142.6 (4)	O3—N2—C14—C15	−127.7 (5)
O1—Sn1—O3—N2	−161.5 (4)	C20—N2—C14—C19	−137.1 (6)
C28—Sn1—O3—N2	−52.6 (4)	O3—N2—C14—C19	50.1 (7)
C27—Sn1—O3—N2	99.7 (4)	C19—C14—C15—C16	1.2 (9)
O2—Sn1—O3—N2	−148.7 (3)	N2—C14—C15—C16	178.9 (5)
O4—Sn1—O3—N2	26.3 (3)	C14—C15—C16—C17	−0.7 (10)
O3—Sn1—O4—C20	−22.4 (4)	C15—C16—C17—C18	−0.9 (11)
O1—Sn1—O4—C20	−35.1 (5)	C16—C17—C18—C19	2.1 (11)
C28—Sn1—O4—C20	82.2 (4)	C17—C18—C19—C14	−1.6 (10)
C27—Sn1—O4—C20	−136.1 (4)	C15—C14—C19—C18	−0.1 (9)
O2—Sn1—O4—C20	152.8 (3)	N2—C14—C19—C18	−177.8 (5)
C7—N1—C1—C6	−140.5 (6)	Sn1—O4—C20—N2	14.5 (6)
O1—N1—C1—C6	48.6 (7)	Sn1—O4—C20—C21	−168.1 (4)
C7—N1—C1—C2	42.6 (8)	O3—N2—C20—O4	7.7 (8)
O1—N1—C1—C2	−128.3 (5)	C14—N2—C20—O4	−164.9 (5)
C6—C1—C2—C3	0.6 (9)	O3—N2—C20—C21	−169.9 (5)
N1—C1—C2—C3	177.5 (5)	C14—N2—C20—C21	17.6 (9)
C1—C2—C3—C4	−0.6 (9)	O4—C20—C21—C22	46.8 (8)
C2—C3—C4—C5	−0.7 (10)	N2—C20—C21—C22	−135.7 (6)
C3—C4—C5—C6	1.8 (10)	O4—C20—C21—C26	−131.6 (6)
C2—C1—C6—C5	0.6 (9)	N2—C20—C21—C26	45.9 (8)
N1—C1—C6—C5	−176.4 (5)	C26—C21—C22—C23	−1.1 (10)
C4—C5—C6—C1	−1.8 (9)	C20—C21—C22—C23	−179.5 (6)
Sn1—O2—C7—N1	10.7 (6)	C21—C22—C23—C24	0.0 (12)
Sn1—O2—C7—C8	−172.1 (4)	C22—C23—C24—C25	0.8 (14)
O1—N1—C7—O2	7.7 (8)	C23—C24—C25—C26	−0.4 (13)
C1—N1—C7—O2	−162.8 (5)	C24—C25—C26—C21	−0.7 (11)
O1—N1—C7—C8	−169.5 (4)	C22—C21—C26—C25	1.4 (10)
C1—N1—C7—C8	20.0 (8)	C20—C21—C26—C25	179.9 (6)
